# Nomogram to predict the progression of patients with primary membranous nephropathy and nephrotic syndrome

**DOI:** 10.1007/s11255-021-02859-x

**Published:** 2021-04-28

**Authors:** Lili Liu, Haitao Wang, Ban Zhao, Xin Liu, Ying Sun, Yonghui Mao

**Affiliations:** Department of Nephrology, Beijing Hospital, National Center of Gerontology, Institute of Geriatric Medicine, Chinese Academy of Medical Sciences, Beijing, 100730 People’s Republic of China

**Keywords:** Primary membranous nephropathy, Nephrotic syndrome, Nomogram, Prognosis

## Abstract

**Background:**

The outcome of patients with primary membranous nephropathy (pMN) who present with nephrotic syndrome (NS) is variable and difficult to predict. The goal of this study was to develop a nomogram to predict the risk of progression for specific individuals.

**Methods:**

This retrospective study involved biopsy-proven patients with pMN and NS treated between January 2012 and June 2018. The primary outcome of our investigation was progression, defined as a reduction of estimated glomerular filtration rate (eGFR) that was equal to or over 20% compared with baseline at the end of follow-up or the onset of end-stage renal disease (ESRD). We used backwards stepwise logistic regression analysis to create a nomogram to predict prognosis. The model was validated internally using bootstrap resampling.

**Results:**

A total of 111 patients were enrolled. After a median follow-up of 40.0 months (range 12–92 months), 18.9% (21/111) patients showed progression. Backwards stepwise selection using the Akaike information criterion (AIC) identified the following four variables as independent risk factors for progression, which were all used in the nomogram: age ≥ 65 years [odds ratio (OR) 7.004; 95% confidence interval (CI) 1.783–27.505; *p* = 0.005], Ln (sPLA2R-Ab) (OR 2.150; 95% CI 1.293–3.577; *p* = 0.003), Ln (proteinuria) (OR 5.939; 95% CI 1.055–33.436; *p* = 0.043) and Ln (Uα1m/Cr) (OR 2.808; 95% CI 1.035–7.619; *p* = 0.043). The discriminative ability and calibration of the nomogram revealed good predictive ability, as indicated by a C-index of 0.888 (95% CI 0.814–0.940) and a bootstrap-corrected C-index of 0.869; calibration curves were also well fitted. A receiver operating characteristic (ROC) curve for the nomogram score revealed significantly better discrimination than each of the three risk factors alone, including Ln (sPLA2R-Ab) [area under the curve (AUC) 0.769], Ln (proteinuria) (AUC 0.653) and Ln (Uα1m) (AUC 0.781) in the prediction of progression (*p* < 0.05). The optimal cutoff value of the nomogram score was 117.8 with a positive predictive value of 44.4% and a negative predictive value of 98.5%.

**Conclusion:**

The nomogram successfully achieved good predictive ability of progression for patients with pMN who present with NS. It can therefore help clinicians to individualize treatment plans and improve the outcome of pMN.

**Supplementary Information:**

The online version contains supplementary material available at 10.1007/s11255-021-02859-x.

## Introduction

Primary membranous nephropathy (pMN) is an autoimmune glomerular disease and one of the leading causes of nephrotic syndrome in adults. In China, the proportion of patients with pMN and primary glomerulopathy increased from 16.8% between 2003 and 2007 to 29.2% between 2008 and 2012 in all groups, and it increased from 53.21 to 70.23% in patients aged ≥ 60 years [1]. MN is characterized by striking granular aggregations of immunoglobulin G (IgG) and electron-dense deposits along the subepithelial aspect of the glomerular basement membrane [2]. Recent breakthroughs indicate that M-type phospholipase A2 receptor (PLA2R), a transmembrane protein located on podocytes, acts as a major antigenic target in pMN. Studies have shown that serum PLA2R antibodies (sPLA2R-Abs) were present in 52–86% of patients with MN, with a specificity of 96–99% for pMN [3–7].

The clinical course and outcome of pMN are variable. Patients with subnephrotic proteinuria usually have excellent long-term renal survival. However, prognosis tends to vary among patients with proteinuria within the nephrotic range. Spontaneous remission of nephrotic syndrome occurs in approximately 20–25% of cases; however, approximately 30% of patients proceed to end-stage renal disease (ESRD) after 10 years [8]. Therefore, it is vital that we identify methods to determine risk factors for renal outcome for patients with pMN who present with NS. For many years, the recommended standard for treating such patients was to evaluate the extent of proteinuria; according to the 2012 Kidney Disease: Improving Global Outcomes (KDIGO) guidelines, patients with > 6 months of proteinuria (> 4 g/day) should receive immunosuppression [9]. However, this risk stratification lacks specificity, as a substantial proportion of such patients may still go into spontaneous remission [10]. Thus, there is an urgent need to identify sensitive and specific predictors that can be used alongside the evaluation of proteinuria. Previous studies demonstrated that the excretion of urinary IgG or low-molecular-weight (LMW) proteins including β2-microglobulin (β2m), α1-microglobulin (α1m), and retinol binding protein (RBP) could predict the disease outcome of patients with pMN [11–14]. Two recent investigations suggested that sPLA2R-Ab is a useful biomarker for predicting prognosis and guiding treatment in pMN patients [15, 16]. Higher levels of this antibody are associated with a lower chance of spontaneous or immunosuppressive therapy-induced remission and a higher risk of renal function deterioration.

Although several clinical parameters and biomarkers have been identified as predictors for pMN, these data were derived from separate studies; there is currently no multivariate predictive model for pMN. In 1997, the Toronto Risk Score was proposed as a useful means of predicting renal outcome in patients with pMN and included three parameters: time-averaged proteinuria (highest sustained proteinuria over a 6-month period), creatinine clearance (CCr) at diagnosis, and the slope of CCr over a 6-month period [17]. Nevertheless, this model was developed some time ago and did not consider the more recently identified predictors including sPLA2R-Ab and urinary biomarkers. Predictive models that include both traditional and new parameters may enable a much more informative assessment of outcome in patients with pMN.

We carried out this retrospective study using a cohort of patients with pMN who presented with NS. Our aim was to develop a novel prognostic nomogram and a prognostic score for renal outcome using a broad spectrum of clinical, laboratory, and pathological parameters available at baseline. We expect that this nomogram could be used to accurately and conveniently predict the progression of patients with pMN and thereby guide clinicians to optimize treatment strategy.

## Materials and methods

### Study population

We conducted a retrospective study on a cohort of pMN patients who underwent native renal biopsy at Beijing Hospital between January 2012 and June 2018. The inclusion criteria were nephrotic syndrome (proteinuria ≥ 3.5 g/day and serum albumin ≤ 30 g/L) and an eGFR ≥ 30 mL/min/1.73 m^2^. Our exclusion criteria were as follows: (1) patients with secondary MN, including autoimmune diseases (lupus nephritis and Sjögren syndrome), infection-related MN (hepatitis B virus-associated MN, hepatitis C virus-associated MN, human immunodeficiency virus-associated MN, and syphilis, MN correlated to malignancies or exposure to toxic agents); (2) a follow-up duration of less than 1 year; (3) the use of immunosuppressive drugs within 3 months prior to kidney biopsy (Supplementary Fig. 1). Baseline and follow-up data were acquired from hospital medical records. Treatment options were in compliance with the KDIGO Guideline for glomerulonephritis.

All study procedures were conducted according to the 2008 Declaration of Helsinki and good clinical practice guidelines. The study was approved by the Ethics Committee of Beijing Hospital (Reference number: 2018BJYYEC-140-1).

### Data collection

Baseline data were collected at the time of renal biopsy, including age, gender, history of hypertension, history of diabetes, hemoglobin (Hb), albumin (ALB), serum creatinine (SCr), blood urea nitrogen (BUN), uric acid (UA), total cholesterol (TCH), triglyceride, low-density lipoprotein cholesterol (LDL-C), high-density lipoprotein cholesterol (HDL-C), immunoglobulin G (IgG), 24-h proteinuria (24hUpro), urinary IgG corrected by creatinine (UIgG/Cr), urinary transferrin corrected by creatinine (UTf/Cr), urinary α1-microglobulin corrected by creatinine (Uα1m/Cr) and *N*-acetyl-β-d-glucosaminidase corrected by creatinine (NAG/Cr). Estimated glomerular filtration rate (eGFR in mL/min/1.73 m^2^) was calculated by the equation put forward by the Chronic Kidney Disease Epidemiology Collaboration (CKD-EPI) and was categorized according to the KDIGO 2012 Clinical Practice Guideline.

Serum was collected from each patient at the time of renal biopsy and stored at − 80 °C. These samples were subsequently thawed to allow serum PLA2R antibody (sPLA2R-Ab) levels to be determined by a commercially available enzyme-linked immunosorbent assay (ELISA) kit (EUROIMMUN AG, Lubeck, Germany). In accordance with the manufacturer guidelines, sPLA2R-Ab levels ≥ 20 RU/mL were considered positive.

For all patients, kidney biopsy was performed at the time of diagnosis. Pathological kidney examination included light microscopy, immunofluorescence, and electron microscopy. Direct immunofluorescence analysis was performed on frozen sections to detect IgG, IgA, IgM, C3, C4, and C1q. Glomerular MN lesions were classified into four stages (I, II, III and IV) based on Ehrenreich and Churg’s criteria. A number of characteristics were evaluated, including the presence or absence of focal segmental glomerular sclerosis (FSGS), acute tubular injury (ATI), and vascular hyalinosis (VH). The degree of tubular atrophy (TA) and interstitial fibrosis (IF) were rated on a scale of 0, 1, or 2 based on the percentage of affected tubules or the extension of IF (< 25, 25–50, > 50%), respectively.

### Outcome

Our primary outcome was progression. This was defined as a reduction of estimated glomerular filtration rate (eGFR) that was equal to or over 20% compared with baseline at the end of follow-up or the onset of ESRD; this definition was in accordance with the risk stratification set out by the 2019 KDIGO Controversies Conference Report [18]. Follow-up time was from the time of renal biopsy to one of three events: ESRD, loss to follow-up, or end of the study (31 December 2019).

### Statistical analysis

Categorical variables are reported as whole numbers and proportions while continuous variables are reported as medians with interquartile ranges (IQRs). Clinical and laboratory variables that are associated with progression risk were assessed a priori based on clinical importance, scientific knowledge, and predictors that were identified in previously published articles. The associations of relevant variables with progression were assessed using a logistic regression model. Backwards stepwise selection using the Akaike information criterion (AIC) was used to identify variables to be incorporated into the multivariable logistic regression model. Odds ratios (ORs) were presented with their 95% confidence intervals (CIs). Selected variables were incorporated into the nomogram to predict the probability of progression using statistical software (rms in R, version 4.0.2; http://www.R-project.org).

Model accuracy was verified using two parameters: discrimination and calibration. The predictive performance and discrimination ability of the nomogram were measured using the concordance index (C-index). The C-index estimates the probability of concordance between predicted and observed outcomes in rank order and is equivalent to the area under the receiver operating characteristic (ROC) curve. Generally, a C-index ≥ 0.70 is suggestive of a good fit. Calibration was evaluated using a calibration plot (a graphic representation of the relationship between the observed outcome frequencies and the predicted probabilities) with a bootstrapped sample of the study group. In a well-calibrated model, the predictions should fall on a 45° diagonal line. Internal validation of the final model was performed with the 1000 bootstrap sample procedure to calculate a C-index that incorporated relative correction.

Finally, the nomogram was used to calculate the total scores for each patient. ROC curve analysis was used to identify the optimal cutoff value that was determined by maximizing the Youden index. The accuracy of the optimal cutoff value was assessed by sensitivity, specificity, and predictive values, and by likelihood ratio.

In all analyses, tests were two-sided, and *p* < 0.05 was considered to indicate statistical significance. All analyses were performed using SPSS, version 25.0 (IBM Corp., Armonk, NY, USA) and R version 4.0.2 (Foundation for Statistical Computing, Vienna, Austria; http://www.R-project.org).

## Results

### Baseline clinical characteristics and outcome

Baseline clinical characteristics and outcome data for the 111 patients enrolled on this study are given in Table 1. The median patient age was 57 years (IQR 41–66 years), and 61.3% (68/111) of the patients were male. At the time of kidney biopsy, patients presented with a median eGFR of 99.2 mL/min/1.73 m^2^ and proteinuria of 5.7 g/day. Eighty-one (73.0%) patients were positive for sPLA2R-Ab as determined by ELISA, with a cutoff value of 20 RU/mL; median level of sPLA2R-Ab was 212.6 RU/mL (IQR 75.4–416.2). Thirty patients were negative for sPLA2R-Ab with a median level of 5.1 U/mL (IQR 3.5–6.4). The levels of urinary IgG/Cr, Tf/Cr, α1m/Cr, and NAG/Cr were 14.7 mg/g (IQR 8.7–29.1), 2.2 mg/g (1.3–4.0), 307.5 mg/mg (IQR, 207.7–469.9), and 37.1 U/g (IQR 24.5–49.0), respectively.

**Table 1:** Baseline clinical characteristics and outcomes of the pMN patients investigated in this study

Parameters	*n* (%) or median (IQR) (*n* = 111)
Sex, male/female	68 (61.3)/43 (38.7)
Age, years	57 (41–66)
Diabetes, *n* (%)	13 (11.7)
Hypertension, *n* (%)	55 (49.5)
Systolic blood pressure, mmHg	132.0 (123.0–140.0)
Diastolic blood pressure, mmHg	80.0 (720–85.0)
Albumin, g/L	26.0 (23.0–29.0)
Serum creatinine, μmol/L	67.0 (56.0–80.0)
eGFR, mL/min/1.73 m^2^	99.2 (85.9–114.6)
CKD stage, *n* (%)	
1	77 (69.4)
2	28 (25.2)
3	6 (5.4)
sPLA2R-Ab positive, *n* (%)	81 (73.0)
Levels of sPLA2R-Ab, RU/mL	85.9 (10.8–349.0)
Hemoglobin, g/L	132.0 (119.0–144.0)
Serum IgG, mg/dL	601 (433.5–738.0)
Total cholesterol, mmol/L	7.2 (6.1–8.5)
Triglyceride, mmol/L	2.3 (1.5–3.2)
HDL-C, mmol/L	1.2 (1.1–1.6)
LDL-C, mmol/L	4.5 (3.8–5.7)
Proteinuria, g/24 h	5.7 (4.5–7.2)
Urinary IgG/Cr, mg/g	14.7 (8.7–29.1)
Urinary Tf, mg/g	2.2 (1.3–4.0)
Urinary α1m/Cr, mg/mg	307.5 (207.7–469.9)
Urinary NAG/Cr, U/g	38.6 ± 18.6
Kidney pathology, *n* (%)	
IF IgG (+)	111 (100)
IF IgA (+)	18 (16.2)
IF IgM (+)	32 (28.8)
IF C3 (+)	93 (83.8)
IF C1q (+)	25 (22.5)
MN stage, *n* (%)	
I	51 (45.9)
II	45 (40.5)
III	6 (5.4)
Obsolescent glomeruli, %	2.1 (0–6.7)
Focal segmental glomerular sclerosis, *n* (%)	20 (18.0)
Acute tubular injury, *n* (%)	23 (20.7)
Tubular atrophy	
Stage 0	93 (83.8)
Stage 1	18 (16.2)
Interstitial fibrosis	
Stage 0	92 (82.9)
Stage 1	19 (17.1)
Vascular hyalinosis, *n* (%)	54 (48.65)
Immunosuppressive therapy, *n* (%)	79 (71.2)
Complete remission, *n* (%)	59 (53.2)
Partial remission, *n* (%)	31 (27.9)
Progression, *n* (%)	21 (18.9)
ESRD, *n* (%)	11 (9.9)
Follow-up time, months	40.0 (25.0–58.0)

*CKD* chronic kidney disease, *eGFR* estimated glomerular filtration rate, *ESRD* end-stage renal disease, *HDL-C* high-density lipoprotein cholesterol, *IF* immunofluorescence *IQR* interquartile range, *LDL-C* low-density lipoprotein cholesterol, *α1m* α1-microglobulin, *MN* membranous nephropathy, *NAG* *N*-acetyl-β-d-glucosaminidase, *sPLA2R-Ab* serum phospholipase A2 receptor antibody, *Tf* transferrin

Based on kidney biopsy, MN stages I, II, and III, were identified in 45.9%, 40.5%, and 5.4% of patients, respectively. Obsolescent glomeruli were present in 2.1 (0–6.7) of glomeruli, 18.0% of patients (20/111) had FSGS, 48.65% of patients (54/111) had VH, and 20.7% of patients (23/111) had ATI. TA was graded as stage 0 in 93 (83.8%) patients, stage 1 in 18 (16.2%) patients and stage 2 in 0 patients (0%), while IF was rated as stage 0, 1, and 2, in 92 (82.9%) patients, 19 (17.1%) patients, and 0 (0%) patients, respectively.

After a median follow-up of 40 months (range 12–92 months), 18.9% (21/111) of patients showed progression at the end of follow-up and remained within the nephrotic range of proteinuria. Compared with patients without progression, those with progression were significantly older and had significantly higher levels of sPLA2R-Ab and proteinuria and urinary biomarkers (UIgG/Cr, Uα1m/Cr, UTf/Cr, and NAG/Cr; all *p* < 0.05) but significantly lower eGFR and serum albumin levels (*p* < 0.05) (Supplementary Table 1). However, histological and immunofluorescence analyses revealed that there were no significant differences between the two groups with regard to MN stages, C3 deposits, or the presence of ATI. Furthermore, all of the patients with progression were shown to be positive for sPLA2R-Ab and 95.2% of these patients (20/21) had received standard immunosuppressive treatment during follow-up.

### Model specifications and predictors of progression

sPLA2R and urinary biomarker data were not normally distributed and therefore underwent natural logarithmic (Ln) transformation. Univariate logistic analysis identified that age ≥ 65 years, albumin, CKD stage, Ln (proteinuria), Ln (sPLA2R-Ab), Ln (UIgG/Cr), Ln (Uα1m/Cr), and Ln (NAG/Cr), were all significantly associated with progression (*p* < 0.05). These risk factors were then selected as candidate variables for the final prediction model. Backwards stepwise selection using the AIC and a logistic regression model identified the following four variables that had the strongest association with progression risk: age ≥ 65 years, Ln (proteinuria), Ln (sPLA2R-Ab), and Ln (Uα1m/Cr) (Table 2). Multivariable analysis further identified that age ≥ 65 years (OR 7.004; 95% CI 1.783–27.505; *p* = 0.005), Ln (proteinuria) (OR 5.939; 95% CI 1.055–33.436; *p* = 0.043), Ln (sPLA2R-Ab) (OR 2.150; 95% CI 1.293–3.577; *p* = 0.003), and Ln (Uα1m/Cr) (OR 2.808; 95% CI 1.035–7.619; *p* = 0.043) were each independently associated with outcome.

**Table 2:** Logistic regression model showing the association of different variables with progression

Variable	Univariable analysis		Multivariable analysis	
	OR (95% CI)	*p* value	OR (95% CI)	*p* value
Factors selected				
Age, years				
< 65	1 (reference)		1 (reference)	
≥ 65	4.724 (1.727–12.917)	0.002*	7.004 (1.783–27.505)	0.005*
Ln (proteinuria, g/24 h)	4.408 (1.093–17.774)	0.037*	5.939 (1.055–33.436)	0.043*
Ln (sPLA2R-Ab, U/mL)	1.002 (1.001–1.004)	0.001*	2.150 (1.293–3.577)	0.003*
Ln (Uα1m/Cr, mg/mg)	1.002 (1.001–1.004)	0.001*	2.808 (1.035–7.619)	0.043*
Factors not selected				
Sex				
Male	1 (reference)			
Female	1.745 (0.620–4.917)	0.292		
Hypertension				
Without	1 (reference)			
With	1.150 (0.444–2.976)	0.773		
Diabetes				
Without	1 (reference)			
With	2.118 (0.584–0.768)	0.254		
Albumin, g/L	0.826 (0.729–0.937)	0.003*		
Serum creatinine, μmol/L	1.015 (0.996–1.034)	0.131		
Serum IgG, mg/dL	0.999 (0.996–1.001)	0.232		
CKD stage				
CKD 1	1.0 (reference)			
CKD 2/3	4.121 (1.533–11.080)	0.005*		
Ln (UIgG/Cr, mg/g)	1.022 (1.002–1.043)	0.032*		
Ln (UTf/Cr, mg/g)	2.850 (1.358–5.983)	0.006*		
Ln (NAG/Cr, U/g)	1.041 (1.014–1.068)	0.002*		
MN stage				
Stage I	1.0 (reference)			
Stage II/III	1.167 (0.447–3.042)	0.753		
Acute tubular injury				
Without	1.0 (reference)			
With	1.250 (0.404–3.867)	0.699		
Focal segmental glomerular sclerosis				
Without	1.0 (reference)			
With	1.562 (0.496–4.920)	0.446		
Tubular atrophy				
Stage 0	1.0 (reference)			
Stage 1	1.851 (0.578–5.925)	0.300		
Interstitial fibrosis				
Stage 0	1.0 (reference)			
Stage 1	1.696 (0.535–5.383)	0.370		
Vascular hyalinosis				
Without	1.0 (reference)			
With	1.202 (0.465–3.112)	0.704		
C3 deposits				
Without	1.0 (reference)			
With	2.054 (0.434–9.716)	0.364		

*C3* complement 3, *CKD* chronic kidney disease, *Ln* natural logarithm, *MN* membranous nephropathy, *NAG* *N*-acetyl-β-d-glucosaminidase, *OR* odds ratio, *sPLA2R-Ab* serum phospholipase A2 receptor antibody, *Uα1m* urinary α1-microglobulin, *UTf* urinary transferrin

* *p* < 0.05

### Nomogram and model performance

**Fig. 1:**
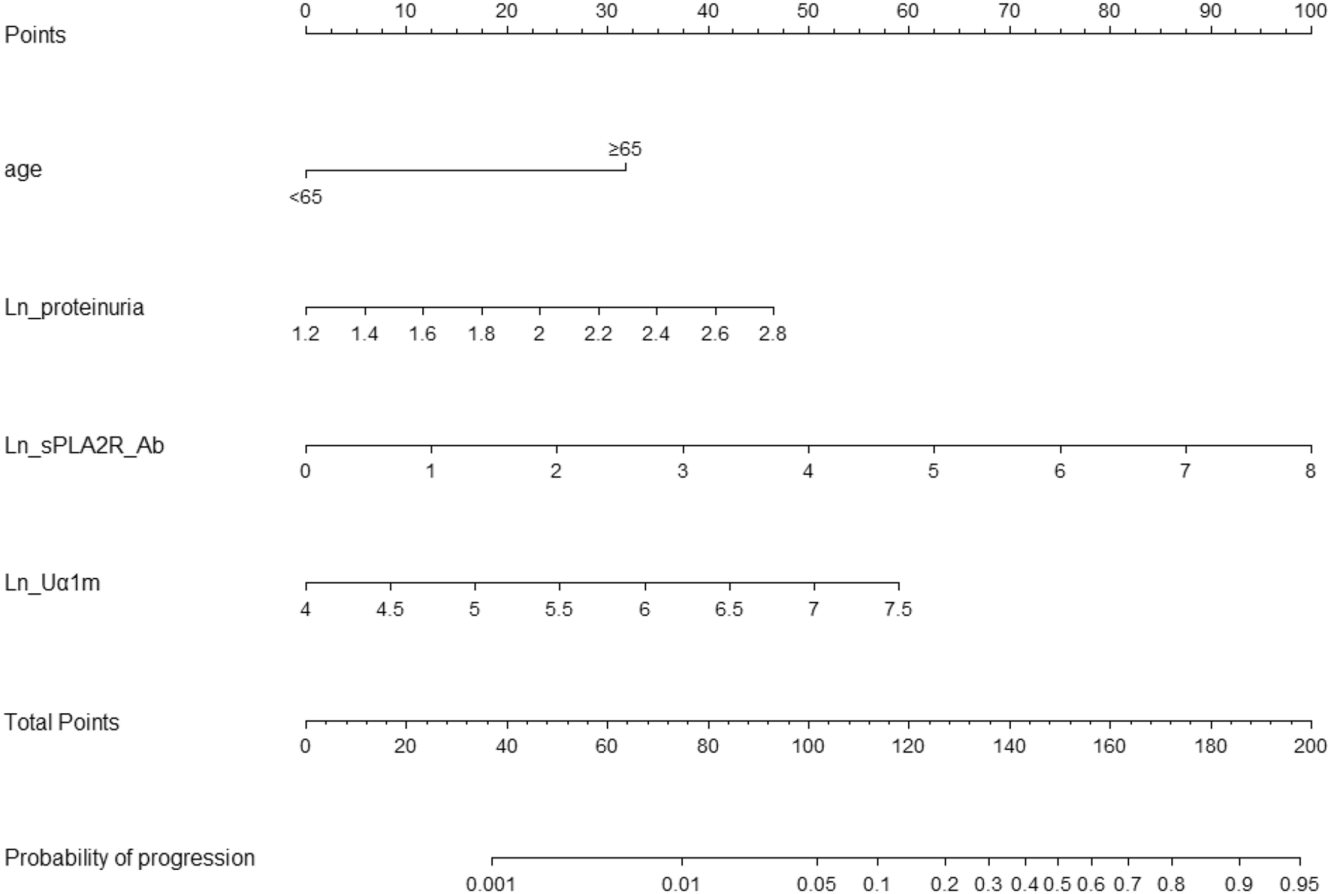
Nomogram predicting progression in pMN patients who present with NS. This nomogram was based on four independent prognostic factors: age ≥ 65 years, Ln (proteinuria), Ln (sPLA2R-Ab) and Ln (Uα1m/Cr)

**Fig. 2:**
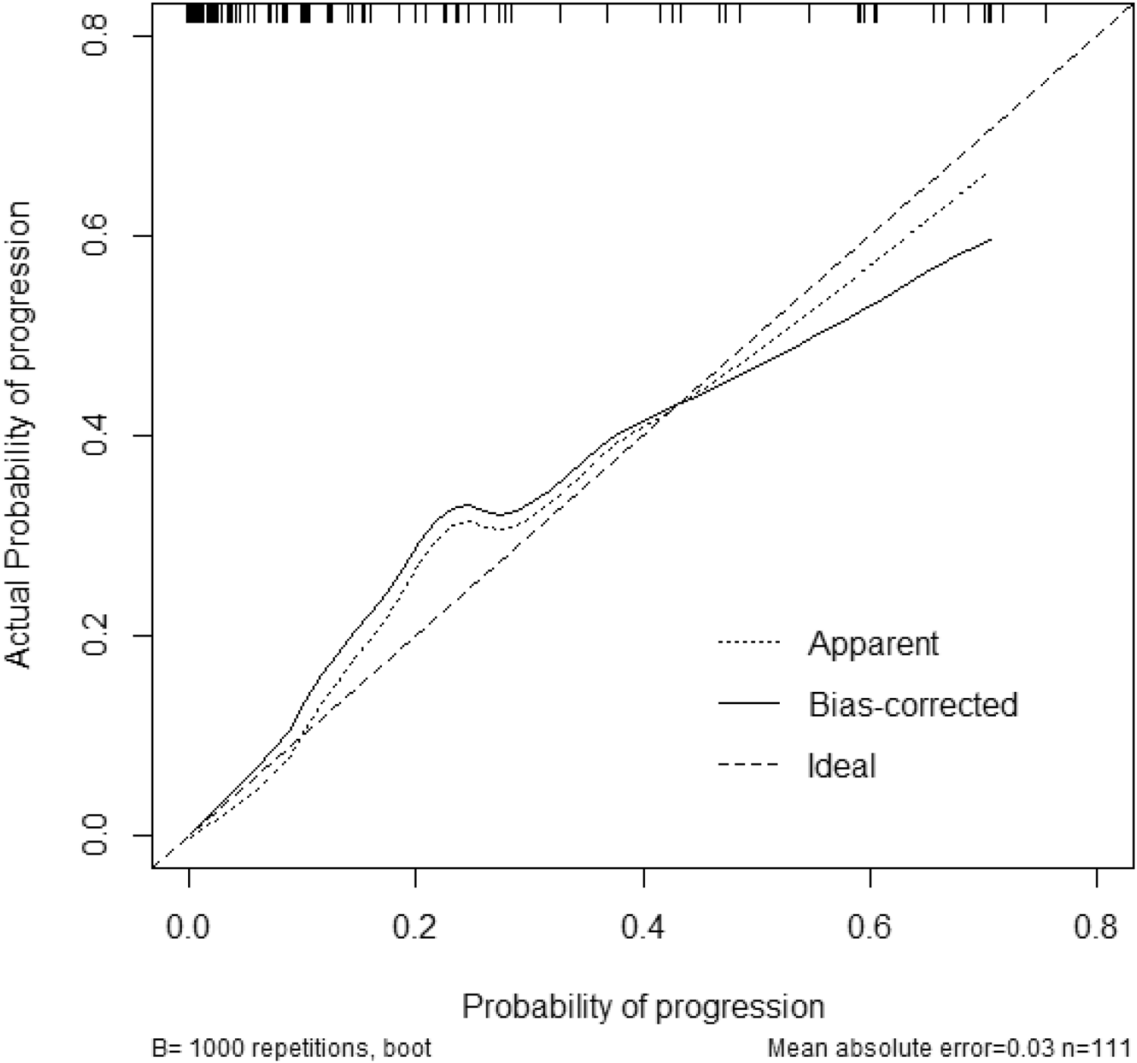
Calibration plot comparing predicted and actual progression probabilities. This shows good agreement with regards to the prediction of progression between the risk estimation by nomogram confirmation

We created a nomogram to predict the progression of patients with pMN who presented with NS (Fig. 1). The nomogram was based on the following four independent prognostic factors: age ≥ 65 years, levels of Ln (proteinuria), levels of Ln (sPLA2R-Ab), and levels of Ln (Uα1m/Cr). A higher total score based on the sum of the assigned number of points for each factor in the nomogram was associated with a worse prognosis. The resulting model was internally validated using the bootstrap validation method. The nomogram demonstrated good levels of accuracy for estimating the risk of progression, with an unadjusted C-index of 0.888 (0.814–0.940) and a bootstrap-corrected C-index of 0.869. The calibration plots revealed good levels of agreement for the prediction of progression and risk estimation, as confirmed by the nomogram (Fig. 2).

**Fig. 3:**
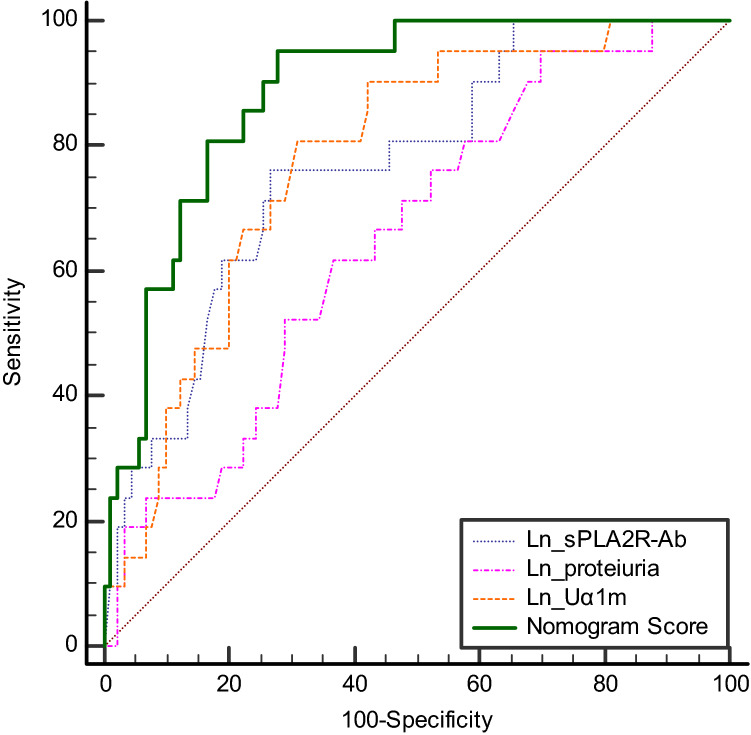
Comparison of ROC curves for progression showing area under the curve (AUC) for the nomogram score (0.888; 95% CI 0.814–0.940), Ln (sPLA2R–Ab) (AUC: 0.769; 95% CI 0.679–0.843), Ln (proteinuria) (0.653; 95% CI 0.557–0.741), Ln (Uα1m) (0.781; 95% CI 0.692–0.854)

In addition, an ROC curve was generated to compare the prognostic values of the identified risk factors with the nomogram scores. The nomogram score [area under the curve (AUC) 0.888, 95% CI 0.814–0.940) was better than each of the three risk factors alone, including Ln_sPLA2R-Ab (AUC 0.769, 95% CI 0.679–0.843), Ln_proteinuria (AUC 0.653; 95% CI 0.557–0.741) and Ln_Uα1m (AUC 0.781; 95% CI 0.692–0.854). This suggested that the risk score had better levels of discrimination for predicting the progression of patients with pMN who presented with NS (Fig. 3).

### Risk of progression based on nomogram scores

The nomogram score for an individual patient was defined as the weighted sum of the individual predictors, with weights equal to the regression coefficients in the final model; scores were calculated as follows: nomogram score = 32 × (1 if age ≥ 65 years) + 29.4 × [Ln (proteinuria) − 1.2] + 12.5 × Ln (sPLA2R-Ab) + 16.9 × [Ln (Uα1m/Cr) − 4.0]. According to the Youden index, the optimal cutoff value for the nomogram score when predicting progression was 117.8. The sensitivity, specificity, positive predictive value, and negative predictive value were 95.2%, 72.2%, 44.4%, and 98.5%, respectively, when considered across the entire cohort (Table 3). Based on the optimal prognostic cutoff value, we categorized all patients into two risk groups: low-risk (*n* = 66; score ≥ 117.8) and high-risk (*n* = 45; score < 177.8). We found that 20 of the 45 patients in the high-risk group showed progression compared to only 1 of the 66 patients in the low-risk group at the end of follow-up (Chi-squared Fisher’s test *p* < 0.001) (Table 4).

**Table 3:** Accuracy of the total nomogram score for predicting progression in pMN patients who presented with NS

Variable	Value (95% CI)
C-index	0.888 (0.814–0.940)
Cutoff score	117.8
Sensitivity, %	95.2 (76.2–99.9)
Specificity, %	72.2 (61.8–81.1)
Positive predictive value, %	44.4 (36.1–53.1)
Negative predictive value, %	98.5 (90.5–99.8)
Positive likelihood ratio	3.43 (2.4–4.8)
Negative likelihood ratio	0.066 (0.010–0.400)

**Table 4:** Association between the risk stratification of the nomogram score and outcome in pMN patients who presented with NS

	Low-risk group (*n* = 66)	High-risk group (*n* = 45)	*p* value Fisher’s test
			< 0.001
Without progression, *n* (%)	65 (98.5)	25 (55.6)	
With progression, *n* (%)	1 (1.5)	20 (44.4)	

## Discussion

Nomograms are increasingly used by clinicians to predict disease and involve simple-to-use digital interfaces that increase predictive power by integrating multiple independent predictors. In the current study, we created a nomogram that includes four independent risk factors (older age, sPLA2R-Ab, proteinuria, and urinary α1m) to predict the progression of patients with pMN who present with NS. This model showed a good level of discrimination and had an excellent C-index of 0.888. Furthermore, the model was validated by bootstrap resampling. In addition, this novel nomogram provided us with a formula to calculate an individual’s risk score with an optimal cutoff value of 117.8. This may help clinicians to individualize treatment plans and follow-up strategies. To the best of our knowledge, this is the first available nomogram based on baseline parameters for predicting the outcome of patients with pMN who present with NS.

In our study, we found that older age, sPLA2R-Ab, proteinuria, and urinary α1m were significant independent predictors of renal outcome in pMN patients who present with NS. The association between age and prognosis in pMN patients was analyzed in previous studies. For example, Kim et al. [19] studied 135 Korean patients with pMN and found that older age (> 60 years) was a significant predictor for a lower rate of complete remission and also showed a higher rate of progression to ESRD although the treatment modalities were similar to those of younger patients. A study from China also demonstrated that age was an independent predictor for a combined outcome consisting of renal function progression, ESRD, and death [20]. These findings are consistent with the present results and indicate that older patients with pMN have an unfavorable clinical course and require more active treatment strategies. However, older age has also been associated with the occurrence of infectious complications in patients who had received immunosuppressants [21]. Collectively, these results suggest that treatment strategy should be individualized in older patients to balance both risks and benefits.

We also confirmed a significant association between baseline sPLA2R-Ab levels and renal outcome in pMN patients who present with NS. These findings are consistent with previous studies [22–25]. However, the precise threshold for the predictive value of sPLA2R-Ab remains controversial. In the extended Evaluate Rituximab Treatment for Idiopathic Membranous Nephropathy (GEMRITUX) study, investigators suggested that a baseline PLA2R-Ab level < 275 RU/mL was independently associated with complete or partial remission of proteinuria at 6 months [10]. In another study involving pMN patients after rituximab treatment, the probability of achieving clinical remission progressively decreased from the lowest tertile (14–86 RU/mL) to the middle tertile (87–204 RU/mL) and the highest tertile (> 204 RU/mL) [26]. The differences between these studies may be attributed to the method of detection, ethnicity, baseline renal function, the ratio of patients with nephrotic-range proteinuria, treatment strategies, and the outcome definition. Therefore, considering the wide distribution of sPLA2R-Ab levels in clinical practice, sPLA2R-Ab was recognized as a continuous variable in our predictive nomogram and could provide more accurate information.

A previous study reported that persistent high-grade proteinuria (> 6 months of proteinuria > 8 g/day) was associated with a high risk of progressive loss of kidney function in patients with membranous nephropathy according to the 2019 KDIGO Controversies Conference Report [18]. However, risk stratification of the degree of proteinuria alone lacks specificity; measures of urinary protein composition may be a better method. In the present study, we measured levels of urinary IgG, transferrin, and α1m corrected by creatinine in a single urine sample, thus representing high-, medium- and LMW protein, respectively. Univariable analysis further showed that all of these urinary biomarkers were associated with progression in pMN patients who present with NS. Nevertheless, multivariable analysis indicated that only α1m was independently associated with renal outcome. Similar to β2-microglobulin, α1m is also a LMW protein and a known marker of tubulointerstitial injury. The results from two separate studies demonstrated that urinary α1m could replace β2-microglobulin in the prediction of renal failure for pMN patients [13, 27]. Branten et al. [13] observed that urinary IgG was a useful marker for the severity of glomerular damage, and that urinary IgG excretion ≥ 250 mg/24 h had a robust relationship with renal survival in patients with pMN. However, our data were not able to validate the independent correlation between urinary IgG and renal outcome. The reason for this discrepancy may be due to potential linear relationships between different predictive parameters.

In a recent study, Stangou et al. [28] investigated a large cohort of 752 patients with pMN over a long-term follow-up period (112–376 months). These authors found that the presence of FSGS and the degree of TA were significant independent parameters for the prediction of renal function outcome, as determined by multiple regression analysis. However, we did not identify any histological renal parameters that could independently predict the outcome of patients with pMN who present with NS. The differences in these results may be partly due to the relatively lower presence of FSGS and a milder degree of tubulointerstitial injury in our current patients. This also emphasizes the significance of monitoring early measurements of sPLA2R-Ab and urinary biomarkers, as these may be more important than histological changes when predicting outcome. In addition, ATI was observed in 20.7% (23/111) patients; we failed to identify any correlation between these histological changes and renal outcome in our cohort or any other studies. These findings suggest that the long-term renal outcome of pMN is more related to the presence and extent of chronic tubulointerstitial injury rather than ATI or glomerular pathology.

To date, we have had very limited options with regard to a predictive model that is capable of integrating multiple pMN risk factors. Cattran et al. [17] proposed a predictive model for renal prognosis in pMN patients based on dynamic changes of proteinuria and CCr. Another group from China proposed a risk score based on baseline age, proteinuria, and eGFR to predict adverse outcomes in patients with pMN [20]. In the present study, we found that no serum or urinary biomarkers (e.g., sPLA2R-Ab, urinary IgG, or urinary α1m) contributed to the risk score calculation. Our model has several strengths that need to be taken into consideration. First, the two existing models described above were both developed in pMN patients with more extensive clinical manifestations, including both nephrotic- and subnephrotic-range proteinuria, while our study focused on pMN patients with NS who need more attention with regard to treatment selection and prognostication. Second, we identified two important biomarkers (sPLA2R-Ab and Uα1m) as vital parameters that were able to provide early and accurate prediction of renal outcome. Third, we were able to rule out the confounding effects of immunosuppressive treatment on renal outcome because nearly almost all patients showing progression had received immunosuppressive treatment.

Our study also has some limitations that need to be considered. First, the patient cohort was recruited from a single center, and the follow-up period was relatively short. Second, the retrospective design could not exclude all confounding factors. Third, the model lacks external validation. Therefore, further studies with larger sample sizes and longer follow-up times are now needed to validate our predictive model and draw further conclusions.

## Conclusion

We developed an optimized nomogram to provide accurate and early prognosis data for pMN patients with NS, a specific cohort of patients that require more clinical attention with regards to their treatment strategies and prognosis. Using this model, it is possible to determine the risk of a worse outcome for an individual patient. This can help clinicians to individualize treatment and improve the outcome of pMN.

## Supplementary Information

Below is the link to the electronic supplementary material.

Supplementary file1 (DOCX 74 kb)

## Data Availability

All data that support the conclusions of this manuscript are included within the article.

## Abbreviations

AIC: Akaike information criterion
ALB: Albumin
ATI: Acute tubular injury
AUC: Area under the curve
BUN: Blood urea nitrogen
C3: Complement 3
C4: Complement 4
CKD: Chronic kidney disease
CI: Confidence interval
C-index: Concordance index
CCr: Creatinine clearance
Cr: Creatinine
CRP: C-reactive protein
eGFR: Estimated glomerular filtration rate
ELISA: Enzyme-linked immunosorbent assay
EPI: Epidemiology Collaboration
ESRD: End-stage kidney disease
Fib: Fibrinogen
FSGS: Focal segmental glomerular sclerosis
Hb: Hemoglobin
IF: Interstitial fibrosis KDIGO: Kidney Disease Improving Global Outcomes
LDL-C: Low-density lipoprotein cholesterol
LMW: Low molecular weight
HDL-C: High-density lipoprotein cholesterol
IgA: Immunoglobulin A
IgG: Immunoglobulin G
IgM: Immunoglobulin M
IQR: Interquartile range
Ln: Natural logarithm
NAG: *N*-Acetyl-β-d glucosaminidase
NR: Non-remission
NS: Nephrotic syndrome
PLT: Blood platelet count
pMN: Primary membranous nephropathy
PR: Partial remission
RBP: Retinol binding protein
ROC: Receiver operating characteristic
SCr: Serum creatinine
sPLA2R-Ab: Serum phospholipase A2 receptor antibody
TA: Tubular atrophy
TCHO: Total cholesterol
TG: Triglyceride
Uα1m: Urinary α1-macroglobulin
UIgG: Urinary immunoglobulin G
UTf: Urinary transferrin
24hUpro: 24-H proteinuria
VH: Vascular hyalinosis
