# Supplementary material for: Nomogram to predict the progression of patients with primary membranous nephropathy and nephrotic syndrome
Source: Int Urol Nephrol. 2021 Apr 28;54(2):331–41. doi: 10.1007/s11255-021-02859-x (PMC8782821; doi:10.1007/s11255-021-02859-x)

**Supplementary Table 1.** A comparison of patients with and without progression

| **Parameters** | **Without progression**  **n=90** | **With progression**  **n=21** | **p value** |
| --- | --- | --- | --- |
| Age, years | 54.5 (38.0-61.3) | 66.0 (48.5-71.5) | **0.013** |
| Male, n (%) | 53 (58.9) | 15 (71.4) | 0.288 |
| sPLA2R-Ab, RU/mL | 70.1 (6.4-253.3) | 382.3 (152.5- 757.4) | **<0.001** |
| sPLA2R-Ab positive, n (%) | 60 (66.7) | 21 (100) | **0.002** |
| Serum creatinine, μmol/L | 64.0 (52.8-79.3) | 78.0 (67.0-86.0) | **0.009** |
| eGFR, mL/min/1.73 m^2^ | 102.6 (90.0-115.2) | 86.6 (70.2-96.3) | **0.004** |
| CKD stage, n (%) |  |  | **0.006** |
| 1 | 68 (75.6) | 9 (42.9) |  |
| 2 | 17 (18.9) | 11 (52.4) |  |
| 3 | 5 (5.6) | 1 (4.8) |  |
| Albumin, g/L | 25.8±3.4 | 23.0±4.0 | **0.001** |
| Proteinuria, g/24 h | 5.4 (4.2-7.0) | 6.7 (5.1-8.7) | **0.030** |
| Urinary IgG/Cr, mg/g | 12.3 (8.0, 26.4) | 26.1 (15.6, 42.1) | **0.002** |
| Urinary Tf/Cr, mg/g | 1.9 (1.1–3.4) | 4.0 (2.6–5.2) | **0.001** |
| Urinary α1m/Cr, mg/g | 256.5 (180.7, 393.4) | 463.4(355.2, 794.2) | **<0.001** |
| Urinary NAG/Cr, U/g | 33.7 (23.8-45.9) | 43.4 (34.2-69.1) | **0.006** |
| MN stage, n (%) |  |  | 0.929 |
| I | 42 (46.7) | 9 (42.9) |  |
| II | 43 (47.8) | 11 (52.4) |  |
| III | 5 (5.6) | 1 (4.8) |  |
| IF C3 deposits, n (%) | 74 (82.2) | 19 (90.5) | 0.355 |
| Focal segmental glomerular sclerosis, n (%) | 15 (16.7) | 5 (23.8) | 0.443 |
| Acute tubular injury, n (%) | 18 (20.0) | 5 (23.8) | 0.698 |
| Tubular atrophy |  |  | 0.294 |
| Stage 0 | 77 (85.6) | 16 (76.2) |  |
| Stage 1 | 13 (14.4) | 5 (23.8) |  |
| Interstitial fibrosis |  |  | 0.366 |
| Stage 0 | 76 (84.4) | 16 (76.2) |  |
| Stage 1 | 14 (15.6) | 5 (23.8) |  |
| Vascular hyalinosis, n (%) | 43 (47.8) | 11 (52.4) | 0.704 |
| Immunosuppressive therapy, n (%) | 59 (65.6) | 20 (95.2) | **0.007** |

Data were presented as frequencies (percentage) or mean±standard deviation or medians (IQR).

C3, complement 3; CKD, chronic kidney disease; eGFR, estimated glomerular filtration rate; IF, immunofluorescence; α1m, α1-microglobulin; MN, membranous nephropathy; NAG, N-acetyl-β-D-glucosaminidase; sPLA2R-Ab, serum phospholipase A2 receptor antibody; Tf, transferrin.

**Supplementary Figure 1**: Flow chart depicting patient screening and follow-up.


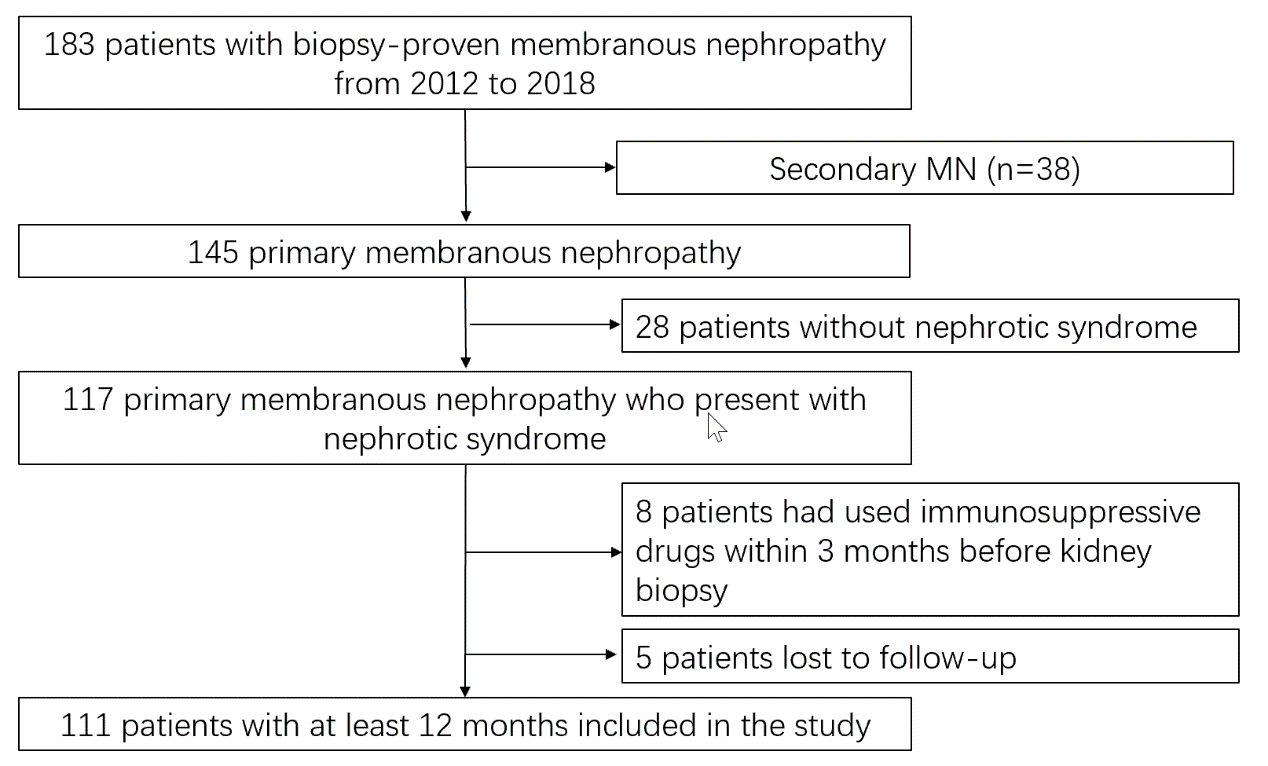

Supplement: Supplementary file 1 — Supplementary file1 (DOCX 74 kb) [file 11255_2021_2859_MOESM1_ESM.docx]
